# Supplementary material for: A set of multi-entry identification keys to African frugivorous flies (Diptera, Tephritidae)
Source: Zookeys. 2014 Jul 24;(428):97–108. doi: 10.3897/zookeys.428.7366 (PMC4143993; doi:10.3897/zookeys.428.7366)
Supplement: Supplementary material 6 — Key to Ceratitis [file zookeys-428-097-s006.zip › SF6_ZooKeys_key to Ceratitis/key/SF6_ZooKeys_key to Ceratitis/Media/Html/Ceratitis striatella.htm]

Ceratitis (Ceratalaspis) striatella Munro


***Ceratitis*** **(*Ceratalaspis*)
*striatella* Munro**

*Ceratitis* (*Pardalaspis*)
*striatella* Munro, 1935: 310.

Body
length: 6.64 (5.00-7.50) mm; wing length: 6.21 (5.80-6.90) mm.

Male

Head.
Antenna yellow-orange. Third antennal segment three times as long as second
segment. Arista with short hairs over entire length. Frons yellow to orange;
with short scattered hairs which are distinctly darker than the frons; concave
to flat, in lateral view not distinctly projecting forwards at antennal
implant. Face yellow-orange. Darker, more orange patches at lower eye margin,
continued along lower margin of face. Chaetotaxy normal for subgenus.

Thorax.
Postpronotum yellow, slightly paler than ground colour of mesonotum, with black
spot. Ground colour of mesonotum mainly yellow-orange, sometimes more greyish.
Chaetotaxy normal for subgenus. Scapular setae pale (in holotype of *striatella*
black except one inner seta pale). One or two anepisternal bristles. Scutellum
yellow with three black separate markings apically; basally two dark separate
spots. Subscutellum pale with three dark spots, widely separate.

Legs
yellow; setation typical for subgenus, mainly pale especially on femora.
Posterior and posterodorsal rows on fore femur pale. Ventral spines black.

Wing
bands with markings extensively yellow. Banding, marginal band continuous;
cubital band free (at most weakly connected with discal band); medial band
absent; crossvein r-m before middle of discal cell. Crossvein dm-cu posteriorly
more outwards than anteriorly.

Abdomen.
Yellow colour. Setation and banding typical for subgenus.

Female.

As
male except for the following� characters: Frons sometimes weakly projected
forwards at antennal implant. Crossvein r-m variable. Oviscape very long, at
least twice as long as abdominal terga 3-6 combined.

(Description
after De Meyer, 1998)
